# Consumer acceptance of and willingness to pay for food nanotechnology: a systematic review

**DOI:** 10.1007/s11051-015-3270-4

**Published:** 2015-11-30

**Authors:** Emma L. Giles, Sharron Kuznesof, Beth Clark, Carmen Hubbard, Lynn J. Frewer

**Affiliations:** Health and Social Care Institute, Teesside University, Constantine Building, Middlesbrough, North Yorkshire TS1 3BA UK; School of Agriculture, Food and Rural Development, Newcastle University, Agriculture Building, Newcastle upon Tyne, Tyne and Wear NE1 7RU UK

**Keywords:** Nanotechnology, Consumer, Acceptance, Expert opinion, Systematic review

## Abstract

Consumer’s attitudes to, and acceptance of, emerging technologies and their applications, are important determinants of their successful implementation and commercialisation. Understanding the range of socio-psychological, cultural and affective factors which may influence consumer responses to applications of nanotechnology will help “fine-tune” the development of consumer products in line with their expectations and preferences. This is particularly true of applications in the food area, where consumer concerns about technologies applied to food production may be elevated. This research applied systematic review methodology to synthesise current knowledge regarding societal acceptance or rejection of nanotechnology applied to agri-food production. The objective was to aggregate knowledge derived from different research areas to gain an overall picture of consumer responses to nanotechnology applied to food production. Relevant electronic databases of peer-reviewed literature were searched from the earliest date available, for peer-reviewed papers which reported primary empirical data on consumer and expert acceptance of agri-food nanotechnology, using a formal systematic review protocol. Inclusion criteria for papers to be included in the review were: empirical peer-reviewed papers written in English; a population sample of adults aged 18 years and over used in the research; a research focus on consumer and expert acceptance of agri-food nanotechnology; and research on attitudes towards, and willingness to pay for, different applications of agri-food nanotechnology. Two researchers independently appraised the papers using NVivo 10 QSR software. Studies examining consumer and expert acceptance were thematically analysed, and key information was collated. The results were synthesised in order to identify trends in information relevant to consumer acceptance of nanotechnology applied to food production. Eight key themes were identified from the 32 papers which were extracted from the literature. These themes were applied to understand the determinants of consumer acceptance of agri-food nanotechnology. Nanotechnology is more likely to be accepted by consumers when applied to development of novel packaging with distinct benefits rather than when integrated directly into agri-food products. Trust and confidence in agri-food nanotechnology and its governance need to be fostered through transparent regulation and development of societally beneficial impacts to increase consumer acceptance.

**Electronic supplementary material:**

The online version of this article (doi:10.1007/s11051-015-3270-4) contains supplementary material, which is available to authorised users.

## Background

There has been extensive debate about the potential societal responses to (different) applications of nanotechnology primarily because consumer’s attitudes towards, and acceptance of, emerging technologies, and their applications are important determinants of their successful implementation and commercialisation, and without consumer acceptance the potential economic and social benefits of nanotechnology may not be realised (Burri and Bellucci 2008; Frewer et al. 2011; Gupta et al. 2011; Kim et al. 2014; Lowe et al. 1993; Macoubrie 2006; Pidgeon et al. 2011; Renn and Roco 2006; Roco 2003). Stakeholders (drawn from industry and policy communities) have identified applications in the agri-food sector as being the potentially most controversial as far as societal acceptance is concerned (Gupta et al. 2013; Matin et al. 2012). To some extent this reflects expert perceptions that the pattern of societal response to different applications of nanotechnology will be similar to those observed following the introduction of genetically modified (GM) foods (Gupta et al. 2015; Mehta 2004). To date however, there has been little evidence of consumer opposition to agri-food applications of nanotechnology, (George et al. 2014), nor has formalised opposition (for example, through activities linked to pressure groups) been as extensive as that associated with GM foods (Seifert and Plows 2014; van Broekhuizen and Reijnders 2011). It is also important to note that attitudes towards technology are unlikely to remain static in space and time, and the results of a single study are unlikely to reflect an aggregated analysis of multiple studies which use different methodologies, study populations, or applications, and which are embedded in different contexts. The aim of this study was to synthesise current knowledge regarding consumer and expert acceptance or rejection of nanotechnology applied to agri-food production, to identify emerging trends and patterns, and to assess gaps in knowledge.

Whilst there have been systematic reviews of the regulatory situation surrounding nanotechnology (Grobe 2008), to the best of the authors’ knowledge, no systematic reviews of research investigating consumer attitudes, perceptions and acceptance of agri-food nanotechnology have been conducted or registered on the PROSPERO^1^ (PROPSERO 2012) database, nor on the databases of the Centre for Reviews and Dissemination (Centre for Reviews and Dissemination 2012; Besley et al. 2008) The systematic reviews that have been conducted to date are in the general area of nanotechnology application (e.g. in relation to risk assessment) or have focused on specific food issues, such as vitamin D food fortification (Black et al. 2011). A systematic review of research into consumer’s attitudes towards and acceptance of agri-food nanotechnology is timely and policy relevant, as simply considering attitudes to specific applications may not reflect general trends in attitudes and consumer priorities for development.

This review seeks to synthesise existing knowledge regarding consumer attitudes towards agri-food nanotechnology in order to provide policy makers, nanotechnology experts, and food manufacturers with robust and high-quality evidence concerning consumer acceptance of nanotechnology applied within the agri-food sector. The results can be applied to providing evidence which will assist key stakeholders in their decision making, facilitate fine-tuning of policies and enable an estimation of how consumers may react to future food products, in line with best practices in agri-food technology application (Cook and Fairweather 2007; Raley et al. 2015).

## Methods

**Fig. 1:**
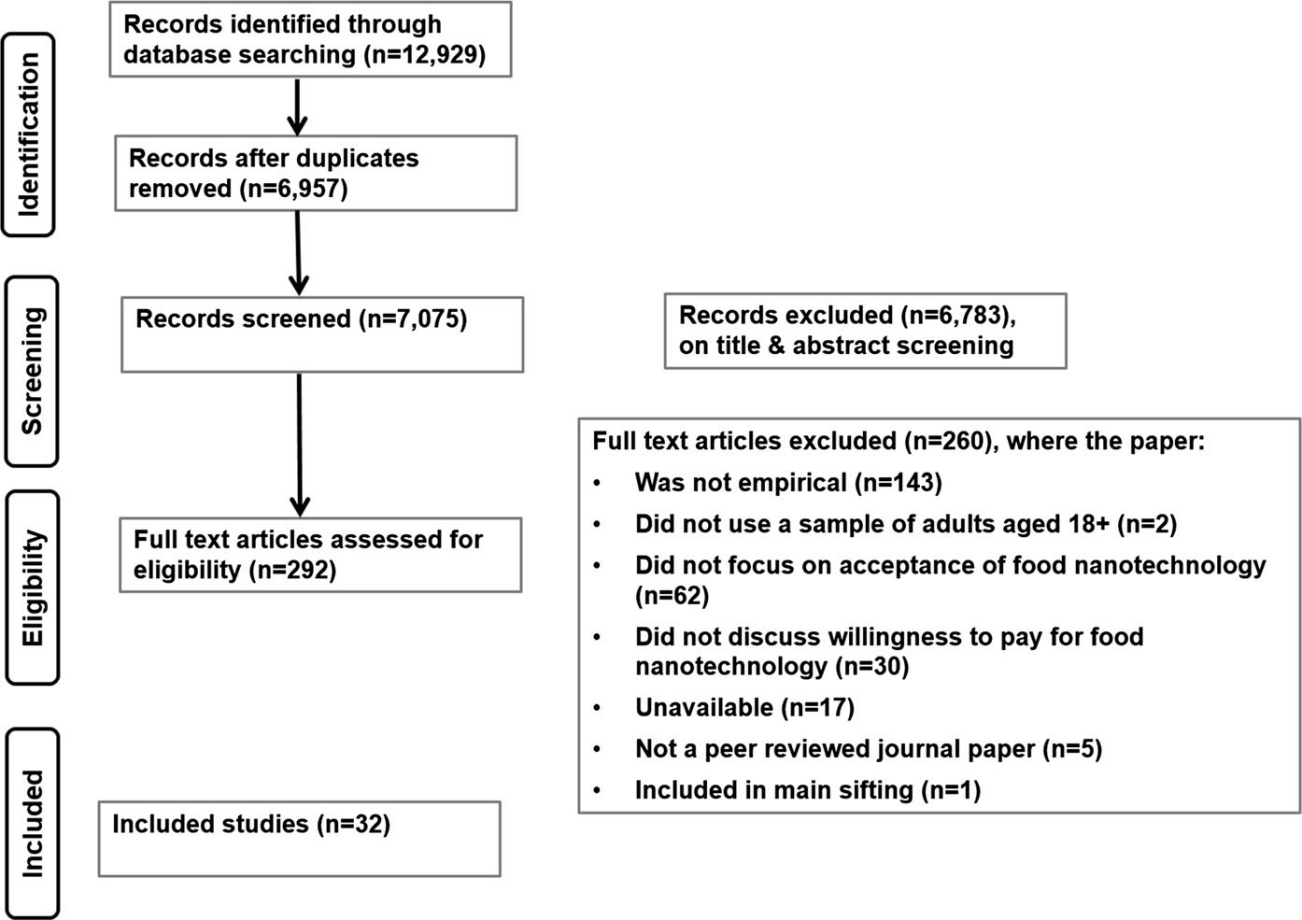
PRISMA flow diagram

A protocol (see Supplementary Data 1) for the review was compiled in full before searching commenced, and there were no substantive variations from protocol during the course of the study. Reporting of the review follows the Preferred Reporting Items for Systematic Reviews (PRISMA checklist; Fig. 1) guidelines (see Supplementary Data 2: Moher et al. 2009).

### Information sources

Seven electronic databases of peer-reviewed literature were searched from the earliest date available (indicated in brackets) to October 2015. These were CAB Abstracts (1910), EBM Reviews (1991), Embase (1980), Medline (1946), PsycINFO (1806), Scopus (1960) and Web of Science (1864). The search strategy combined relevant terms for ‘nanotechnology’, ‘food’ and ‘consumer acceptance’, and search strings were adapted as appropriate for each database. Examples of the search terms used are provided in Supplementary Data 3. Additionally, reference lists of all papers meeting the inclusion criteria were also reviewed, and citation searches of included papers were conducted using Web of Science. Endnote X6 was used to manage search results, with NVivo 10 QSR International software subsequently used for data analysis.

### Eligibility criteria

Studies deemed eligible for inclusion were papers which reported primary empirical data on consumer and expert acceptance of agri-food nanotechnology. Only peer-reviewed papers, written in English, were included in this review in order to focus on high-quality evidence on the acceptance of agri-food nanotechnology. The inclusion criteria are fully described in Table 1 and were established to answer the primary research question: How acceptable is nanotechnology to consumers and experts when applied to agri-food products?

**Table 1:** Inclusion criteria

Study component	Inclusion criteria
Date range	All dates
Publication characteristics	English language, peer-reviewed journal article
Study design	Empirical, qualitative and/or quantitative primary data
Population	Adults aged 18 years and over
Focus	Must contain a discussion of consumer acceptance of food nanotechnology
Outcome	Must contain discussion of willingness to pay/intention to pay for food nanotechnology products

### Study selection, appraisal and synthesis

Papers were screened by two independent researchers (ELG and BC) in a three-stage process in relation to the eligibility criteria. This was done at title, abstract and full text level. Any disagreements were resolved by face-to-face discussion. Due to reference lists and citation searches being conducted, some studies were included which contained the same population as previous studies (Brown et al. 2015; Yue et al. 2015b; Roosen et al. 2011). Where studies report the same data, they are only reported once in the result i.e. there are 32 papers but only 29 stand-alone studies.

Quality assessment of included studies was carried out independently by two researchers (ELG & BC) with the Critical Appraisal Skills Programme Qualitative Research Checklist (Critical Appraisal Skills Programme 2013) used to assess qualitative research. To assess the quantitative papers, the survey research tool by Petticrew and Roberts (2006) was used. For the mixed methods papers, both tools were used for quality appraisal. Disagreements were resolved through discussion (ELG & BC).

The studies examining consumer and expert acceptance are presented in a tabular summary for narrative synthesis (see Table 2). They are described in terms of their aims, methods and study participants along with a brief summary of their key findings. Due to the plethora of findings, inconsistency in reporting styles and complexity and mixed methods nature of the data, studies were deemed too heterogeneous for meta-analysis, a four-stage thematic analysis approach was taken (Braun and Clarke 2006).

**Table 2:** Table of included studies

Paper	Aim	Methods (n)	Participants	Country	Major conclusions
Becker (2013)	To understand how the nanotechnology industry perceives the risks of nanotechnology	Semi-structured, open-ended phone interviews (*n* = 17)	American individuals involved in the commercialization of nanotechnology	USA	Commercialisers acknowledged uncertainty to be inherent to the overall risk arising from nanotechnology and thus take a lot of precaution in ensuring the safety of their products. However, they claim that nanotechnology is neither novel nor risky
Besley et al. (2008)	To provide evidence regarding what American researchers, who have published research on nanotechnology, view as the most important potential benefits and risks of nanotechnology-oriented research, as well as views about the current state of government regulation, the current state of research and its future. It also explores which expert perceptions represent broadly a shared consensus and which provoke a range of individual opinions	Survey (*n* = 177)	Nanotechnology American researchers	USA	Researchers acknowledged the importance of a range of nanotechnologies across a diversity of areas. Health and technological benefits were perceived to be more important than environmental benefits. However, public health and environmental issues are argued to be areas where both risks and the need for regulation are greatest
Bieberstein et al. (2013)	To evaluate consumers’ willingness to pay (WTP) for food nanotechnology focusing on: nano-fortification with vitamins and nano-packaging. Specifically, to evaluate the impact of information on consumer choice when nanotechnology may have important but uncertain consequences on health, environment and society	(Choice) experiment based on sample of 143 German participants, and 152 French participants Sample random sampling using quotas	French and German consumers	France and Germany	Most participants in this study expressed their reluctance to accept nanotechnology applications in food products. Food safety and its link to human health are very important when considering nano-foods. There are differences across the two countries, with French consumers being more reluctant to accept nano-packaging, whereas German consumers are more concerned about nano-fortification
Brown et al. (2015)	To better explore and understand the public’s perceptions of and attitudes towards emerging technologies and food products	Focus groups (*n* = 7) 90 min in length and ranging in size from seven to 10 participants. Participants selected on the criteria an equal number of females and males in each group	56 participants (citizens/public) across 6 US cities	USA	Scepticism and altruism are two factors yet unrecognised as influential in the public’s perceptions of nanotechnology. Hence, they may play an important role in explaining how and why perceptions are formed. These factors also provide a bridge between cultural-based theories and psychometric-based theories
Brown and Kuzma (2013)	To examine public attitudes towards food nanotechnology in conversational, focus group settings, in order to identify policy options for nano-food governance, particularly options for labelling	Focus groups (*n* = 7) 90 min in length and ranging in size from seven to 10 participants. Stratified random sampling. Quantitative worksheet responses, followed by post-group online survey (*n* = 34)	56 participants (citizens/public) across 6 US cities	USA	Participants required nanotechnology labels for all types of food and most of them were willing to pay a premium for labelling. However, labels alone are insufficient to help consumers to make informed choices
Capon et al. (2015)	To develop evidence regarding perceptions of labelling products made by nanotechnology	Representative national cross-sectional household survey (*n* = 1355) using computer-assisted telephone interviewing landline and mobile phone technologies. Random-digit dialled sampling. A similar survey (*N* = 1850) with academic, business and government stakeholders	Australian larger public, academic, business and government stakeholders	Australia	Support for labelling of nano-products is wanted by all stakeholders. However, the public are less likely to buy these products than any other stakeholders
Casolani et al. (2015)	To examine consumers’ acceptance of nanotechnology application in wine production	Representative regional (face-to-face) survey (*N* = 221). Conjoint and post hoc segmentation analysis	Italian wine consumers from the Abruzzo region	Italy	Consumers are relatively unfamiliar with applications of nanotechnology and possess an overall rejection of the concept of “nano-wine”. However, nanotechnology becomes more acceptable when its specific application enhances wine attributes
Cobb and Macoubrie (2004)	To discover the status of US public opinion/concern or interest (knowledge, risk, benefits and trust) in nanotechnology	Representative national phone survey (*N* = 1536). Random-digit dialled survey	Public/citizens—adults 18 years or older in the continental US	USA	American citizens pay scant attention to science in general and nanotechnology in particular, and hence they have minimal knowledge about it. However, respondents who have heard about nanotechnology were more likely to associate it with potential benefits. Emotions (particularly the emotion of feeling hopeful) played an important role in explaining respondents’ attitudes towards nanotechnology
Conti et al. (2011)	To assess public perceptions of nanotechnology by exploring perceived risks (risk versus benefit framings) and the specific social positions from which people encounter or perceive new technologies	National phone survey (*N* = 1100)	American public	USA	Public’s acceptance of nano-enabled products depends on a multitude of factors. Assessments of risks and benefits are strongly linked to the systematically manipulated psychometric qualities of various nanotechnology applications. With some exception, (social) justice plays an important role in the formation of risk perceptions related to nanotechnology
Cook and Fairweather (2007)	To provide an early assessment of key influences on intentions to purchase low fat lamb or beef made using nanotechnology	Focus groups (*N* = 40) to identify beliefs associated with the new food National postal survey (*N* = 565)	New Zealand public	New Zealand	Participants are more likely to purchase low fat lamb or beef made using nanotechnology. The intentions to purchase these products were influenced by self-identity, attitude and subjective norms
Farshchi et al. (2011)	To examine public awareness and attitudes of Iranian people towards nanotechnology, including the role of affect and trust in shaping public opinion on this technology	Survey (*N* = 759)	759 individuals demographically weighted to reflect general population of 16 years and older in Tehran	Iran	The majority of participants were not familiar with the concept of nanotechnology. However, perceived benefits are more likely to outweigh perceived risks. Attitude towards nanotechnology particularly driven by hopes and expectations
Groves (2013)	To examine the prospects (difficulties and opportunities) of nanoscale science and technology commercialisation by implementing adaptive and*/*or anticipatory regulation and to identify potential challenges to its implementation	Policy Delphi (*n* = 13)	A multi-stakeholder panel including individuals from central government and regulatory agencies, consultancies, natural and social academic science and civil society organisations	UK	The panel saw little prospect of a disruptive nanoscale science and technology (NST) future triggered by a radical new technical paradigm. At the strategic level, there is a need for trade-offs between flexibility and resilience. Benefits of NST are perceived particularly for luxury goods manufacturers rather than society at large. Regulators, governments and industry are encouraged to avoid a ‘fast, fragile and fragmented’ future
Gupta et al. (2015)	To elicit the factors that shape consumer perception of different applications of nanotechnology	Structured interviews (*n* = 18 participants). Repertory grid method in conjunction with generalised Procrustes analysis	Consumers from a city (Newcastle upon Tyne) in the North East of England	UK	Consumers differentiate between applications of nanotechnology based on their perceived benefits. However, these may be off-set particularly by perceived risks of fear and ethical concerns
Gupta et al. (2012)	To identify expert opinion on factors influencing societal response to applications of nanotechnology. Specifically, to compare different applications of nanotechnology and identify expert views regarding factors influencing societal acceptability	Structured face-to-face interviews (*n* = 17). Repertory grid methodology in conjunction with generalised Procrustes analysis	Experts on nanotechnology engaged in diverse activities related to nanotechnology, across the North West of Europe	North West of Europe (Germany, Ireland, UK and the Netherlands)	The societal response to different nanotechnology applications depends mainly on the extent to which these applications are perceived to be beneficial, useful and necessary and how ‘real’ and physically close they are to the end-user
Gupta et al. (2013)	To examine differences in expert opinion regarding societal acceptance of different applications of nanotechnology within different technological environments, consumer cultures and regulatory regimes	Online questionnaire designed and administered using Qualtrics software (*n* = 67)	Experts from Northern America (*N* = 12); Europe (*N* = 21); India (*N* = 12); Singapore (*N* = 11) and Australasia (*N* = 11) Academia, industry, government, media and consumer representative groups	Northern America Europe; India Singapore and Australasia	All experts agreed that perceived risk and consumer concerns regarding contact with nanoparticles are more likely to drive rejection, whereas perceived benefits influence acceptance, no matter the country. Encapsulation and delivery of nutrients in food was thought to be the most likely to raise societal concerns, whilst targeted drug delivery was most likely to be accepted. Social acceptance may be homogenous, independent of local contextual factors
Handford et al. (2015)	To assess awareness and attitudes of agri-food organisations towards nanotechnology	Face-to-face and phone interviews (*n* = 14) and an electronic questionnaires administered to a large database (*n* = 1014)	Agri-food organisations	Ireland	Current awareness of nanotechnology applications in the Irish agri-food sector is low. Participants do not have strong (negative or positive) views regarding applications of nanotechnology to this sector
Köhler and Som (2008)	To examine whether innovators, the pioneers of the technological advance in nanotechnology, are aware of the lessons that can be learned from adverse effects that have occurred following past innovation	Interviews (*n* = 20) using structured questionnaires based on the relevant issues identified in the literature review. Most by phone plus some face-to-face questionnaire responses	Innovators/experts (researchers and engineers involved in R&D on nanotechnology-based applications, at both universities and businesses) Nanotechnology application areas: “medical diagnosis”, “food packaging” and “energy conservation and production”; marketing and regulating nanotechnology	12 European countries (no clear specification)	Innovators are less sensitive to early scientific warnings regarding risks of nanotechnology. However, they hardly engage in risk communication and dialogue with stakeholders. Lack of public acceptance of nanotechnology is perceived as a barrier by innovators and many fear a ‘backslash’. Innovators are confident that risks associated with nanotechnology are measureable and manageable
Marette et al. (2009)	To evaluate the impact of information on consumers’ choice (WTP) when nanotechnology may have important but uncertain consequences on health, environment and society	(Choice) experiment (*n* = 97) randomly selected based on quota sampling	German consumers	Germany	The majority of participants are reluctant to accept nanotechnology in food products. Health information is a priority for consumers and the lack of it reduced considerably the WTP for these products
Roosen et al. (2015)	To assess the impact of trust on the willingness to pay for nanotechnology food	Online survey in Canada (*N* = 615) and Germany (*N* = 750) plus an economic laboratory experiment in Germany (*n* = 143)	Larger public/consumers	Canada and Germany	Nanotechnology applications, related to food and drink (juice) and packaging, raise concerns in people’s minds. Trust can lessen these concerns. WTP for nanotechnology increases with trust
Roosen et al. (2011)	To evaluate the impact of different information sequences on participants’ hypothetical WTP for food produced using nanotechnology that may have uncertain consequences for health, the environment, and society	(Choice) experiment (*n* = 143) randomly selected based on the quota method	German consumers	Germany	Information choice plays an important role in assessing impacts of food produced using nanotechnology. Health information clearly decreases WTP, whereas societal and environmental information have a lower effect on WTP. Consumer benefit depends on their perceptions regarding the safety of nanotechnology food products
Schnettler et al. (2013a)	To evaluate acceptance of nanotechnology applications in sunflower oil and in food packaging by consumers in Temuco (Region of the Araucanía, Chile) and identify consumer segments according to their knowledge of nanotechnology, socio-demographic characteristics, and their level of satisfaction with food-related life	Survey (*n* = 400). Simple random sampling	Shoppers (people responsible for buying food for their households)	Chile	Consumers’ perception regarding new food should be considered from an early stage of the product development process. Brand is an attribute which matters relatively more than nanotechnology application in packaging and food. It is also more important than price
Schnettler et al. (2013b)	To investigate the relationship between food neophobia, satisfaction with life and food-related life, and acceptance of the use of nanotechnology in food production	Survey (*n* = 400)	Supermarket shoppers in southern Chile	Chile	The study confirms the existence of a positive relationship between satisfaction with life and satisfaction with food-related life. Four consumers groups were identified. Groups differ in their knowledge of nanotechnology, willingness to purchase foods involving nanotechnology, age, socioeconomic level and lifestyle. The degree of food neophobia is associated with satisfaction with life, with food-related life, as well as with the acceptance of nano-products
Schnettler et al. (2014)	To compare the acceptance of sunflower oil produced with nanotechnology with the acceptance of genetically modified and conventionally produced foods among consumers in Temuco (Region of the Araucanía, Chile), to differentiate market segments according to their acceptance of nanotechnology, and to characterise these segments according to their socio-demographic characteristics and level of food neophobia	Survey (*n* = 400)	Supermarket shoppers in southern Chile	Chile	The majority of respondents had no previous information on nanotechnology or knew its meaning. Brand and production technology were identified as the main attributes that influenced the decision to purchase sunflower oil. This was followed by price and the existence of a health certification seal
Siegrist et al. (2007)	To investigate how lay people perceive nanotechnology foods and nanotechnology food packaging, and examine the factors that influence willingness to buy (WTB) these products	Survey (*n* = 153)/ Convenience sample	Shoppers (persons who are responsible for grocery shopping) from the German-speaking part of Switzerland	Switzerland	Overall, participants were reluctant to buy nanotechnology foods or food with nanotechnology packaging. However, packaging is perceived as more beneficial than nano-foods. Social trust in the food industry directly influences the affect aroused by these new products and WTP. The affect has an impact on perceived benefits and risks. The latter seems to be the most important predictor for WTP
Siegrist et al. (2008)	To examine how lay people perceive various nanotechnology foods and nanotechnology food packaging and to identify food applications that are more likely and food applications that are less likely to be accepted by the public	Mail survey (*n* = 337). Random sampling	Person in the household next in line for their birthday and over 18 years of age in the German-speaking part of Switzerland	Switzerland	Affect and perceived control influence risk and benefit perception of nanotechnology food. Packaging seems to be less problematic than nanotechnology in foods. Naturalness in food products and trust are significant factors that influence the perceived risk and benefit of nanotechnology foods and nanotechnology food packaging
Siegrist et al. (2009)	To examine consumers’ willingness to buy health-beneficial food products produced using nanotechnology	Two representative mail surveys (*n* = 255 & *n* = 260). Random sampling	Person in the household next in line for their birthday and over 18 years of age in the German-speaking part of Switzerland	Switzerland	Consumers were hesitant to accept nano-foods. They attribute a negative utility to nanotechnology foods, even when the food products had clear health benefits for the consumers. Perceived naturalness influences positively the willingness to buy functional foods. Health benefits due to natural additives had a higher utility compared with additives tailored using nanotechnology
Simons et al. (2009)	To analyse the recognition, risk perception and acceptance of nanotechnology, and to address the problems of risk communication on nanotechnology	In-depth interviews (*n* = 50) plus a phone survey (*n* = 1000)	In-depth interviews: participants selected in line with the requirement to cover a broad range of ways of dealing with nanotechnology and information about it. Survey: people aged between 16 and 60 years, registered in public telephone books that include cell phones, who were capable of understanding and answering questions in German	Germany	In Germany, nanotechnology raises expectations and hopes for improvements, particularly in the fields of medicine and environment. The majority of participants are open to nanotechnology, and perceived risk associated with nanotechnology is low
Stampfli et al. (2010)	To examine factors that may influence the acceptance of nanotechnology products in the food domain. Specifically it investigates the influence of risk information on the acceptance of nanotechnology food and food packaging	Representative mail survey (*n* = 514)	The person in the household next in line for their birthday and over 18 years of age	Switzerland	Attitudes towards gene technology was the strongest variable in explaining the variance of perceived risk and perceived benefit of nanotechnology applications. Social trust had also a significant effect on perceived benefit and perceived risk. However, food and packaging applications containing nanoparticles are perceived differently with the latter receiving greater acceptance
Suhaimee et al. (2014)	To evaluate the level of awareness and knowledge (including risks and benefits) about nanotechnology in Malaysia in relation to demographic profiles. The willingness to buy and use nano-based products was also identified specifically on food-related products	Survey (*n* = 309). Random sampling	Visitors of the Malaysia Agriculture, Horticulture and Agrotourism Exhibition 2012	Malaysia	The level of awareness regarding nanotechnology is low in Malaysia relative to the developed countries. Most participants agreed that the perceived benefits exceed the risks and they were willing to buy nanotechnology-based products
Yawson and Kuzma (2010)	To examine and critically analyse the links between consumer acceptance of agri-food nanotechnology and factors such as trust, stakeholders, institutions, knowledge, and human environmental health risks, by using systems mapping	Meta-analysis of the risk perception literature plus experts’ opinions to develop a systems map (*n* = 21), via electronic surveys and/or phone interviews	Experts in agri-food nanotechnology	n/a	Consumer acceptance of agri-food nanotechnology involves a high level of complexity in which to model and understand how decisions are made. Building trust and confidence in an industry that may involve significant risks such as the agri-food nanotechnology industry, governance systems, especially regulatory aspects of governance systems, were pointed out as key factors in consumers’ acceptance of nanotechnology
Yue et al. (2015a, b)	To investigate heterogeneous consumer preferences for nano-food and genetically modified food	Online survey (*n* = 1117) and choice experiment to compare consumer preferences and willingness to pay (WTP) for GM good and nano-food (i.e. rice)	US consumers	USA	Nano-food is preferable to GM food across all participants. Safety benefits, nutrition, taste and environment are important attributes. However, consumers’ preferences are heterogeneous.
Yue et al. (2015a, b)	To explore the relationship between perceptual influences of consumers such as trust in government to manage technologies, risk and benefit attitudes and labelling preferences on consumers’ willingness to buy (WTB) genetically modified and nano-food products	Online representative survey (*n* = 1145) conducted by a professional company (Qualtrics) Structural equation modelling	US consumers	USA	Trust in government to manage GM and nano-foods does not influence labelling preferences. However, trust does influence attitudes towards food technologies. Labelling influences WTP for nano-foods but not GM foods

The first stage involved reading through the papers line-by-line and highlighting relevant data (e.g. a word or a paragraph), to which a code was assigned. These codes were either sociologically constructed. This means that a code was given to the data by the researchers (ELG and BC), which was either a word, sentence or paragraph, and which best reflected the meaning within the data (e.g. safety, lack of testing, too expensive)—or an ‘in vivo’ code—a code which directly copies what was published in the text (Barnett-Page and Thomas 2009). The second stage of the coding process involved examining these initial codes to ensure all data had been thematically analysed (by ELG and BC). The third stage involved sorting the initial codes into broader categories. Here, the researchers (ELG and BC) reflected upon the array of codes and generated broader categories by merging some codes with others, creating new codes, or re-naming or deleting existing codes. The fourth stage involved assigning several themes, which essentially grouped the initial codes into major themes that would help address the research questions. Memo notes were made on how and why these analytical codes were generated by one researcher (ELG), with two further researchers (BC and SK) verifying them. These themes are presented in Table 5, and are discussed in the next section. They are illustrated using representative quotations to illustrate each theme.

## Results

Thirty two papers were included; six qualitative studies (Becker 2013; Brown et al. 2015; Brown and Kuzma 2013; Gupta et al. 2012; Gupta et al. 2015; Köhler and Som 2008), 23 quantitative studies (surveys and experiments) (Besley et al. 2008; Bieberstein et al. 2013; Capon et al. 2015; Casolani et al. 2015; Cobb and Macoubrie 2004; Conti et al. 2011; Cook and Fairweather 2007; Farshchi et al. 2011; Groves 2013; Gupta et al. 2013; Marette et al. 2009; Roosen et al. 2015; Roosen et al. 2011; Schnettler et al. 2013a; Schnettler et al. 2014; Schnettler et al. 2013b; Siegrist et al. 2007; Siegrist et al. 2009; Siegrist et al. 2008; Stampfli et al. 2010; Suhaimee et al. 2014; Yue et al. 2015a, b) and three mixed methods papers (Handford et al. 2015; Simons et al. 2009; Yawson and Kuzma 2010) (see Table 2). During sifting, 17 papers were excluded because they were unavailable from Newcastle University, the Internet or through inter-library loans, or they were unobtainable in English (Ahmadi and Ahmadi 2013; Cheng et al. 2009; Lin et al. 2011; Militaru and Ionescu 2013; Mir 2007; Rakia 1993; Rogers et al. 2013; Schiffeler 2014; Scholl 2013; Siegrist 2007; Stone 2009; Suerdem et al. 2013; Tanaka 1995; Teggatz 2013; Thoenes 1982; Thompson n.d.; Zimmer 2008), but which may have been potentially relevant. The qualitative empirical papers collected data using focus groups (*n* = 2) (Brown et al. 2015; Brown and Kuzma 2013) and interviews (*n* = 4) (Becker 2013; Gupta et al. 2012; Gupta et al. 2015; Köhler and Som 2008). The quantitative empirical papers largely utilised survey methodology (*n* = 20) (Besley et al. 2008; Capon et al. 2015; Casolani et al. 2015; Cobb and Macoubrie 2004; Conti et al. 2011; Cook and Fairweather 2007; Farshchi et al. 2011; Gupta et al. 2013; Roosen et al. 2015; Schnettler et al. 2013a, b; Schnettler et al. 2014; Siegrist et al. 2007; Siegrist et al. 2009; Siegrist et al. 2008; Stampfli et al. 2010; Suhaimee et al. 2014; Yue et al. 2015a, b), one used a survey as part of a Delphi methodology (Groves 2013), and a further three used experiments (Bieberstein et al. 2013; Marette et al. 2009; Roosen et al. 2011). The mixed methods studies combined a survey and interview methods approach (Handford et al. 2015; Simons et al. 2009; Yawson and Kuzma 2010). Study populations were mainly individual members of the public (consumers/shoppers) (*n* = 23) (Bieberstein et al. 2013; Brown et al. 2015; Brown and Kuzma 2013; Casolani et al. 2015; Cobb and Macoubrie 2004; Conti et al. 2011; Cook and Fairweather 2007; Farshchi et al. 2011; Gupta et al. 2015; Marette et al. 2009; Roosen et al. 2015; Roosen et al. 2011; Schnettler et al. 2013a; Schnettler et al. 2014; Schnettler et al. 2013b; Siegrist et al. 2007; Siegrist et al. 2009; Siegrist et al. 2008; Simons et al. 2009; Stampfli et al. 2010; Suhaimee et al. 2014; Yue et al. 2015a, b), ‘experts’ in the area of nanotechnology (*n* = 6) (Besley et al. 2008; Groves 2013; Gupta et al. 2013; Gupta et al. 2012; Köhler and Som 2008; Yawson and Kuzma 2010), agri-food organisations (Handford et al. 2015),‘commercializers’ (*individuals who make deliberate efforts to increase the presence of products on the market that employ nanotechnology or contain nanomaterials”*(Becker 2013); and one study surveyed consumers, academic, business and government stakeholders (Capon et al. 2015).

Quality appraisal of the qualitative studies is shown in Table 3, and the quantitative studies in Table 4. For the qualitative studies, all six papers included a clear statement of the aims of the research and employed a qualitative methodology. The majority of studies had designs appropriate to the aims and objectives, used a suitable recruitment strategy, collected data in a way that was appropriate to the research topic and provided a clear statement of findings. However, the majority of studies did not consider the impact of the relationship between the researcher and the participants, and only two of them explicitly state how they had considered ethical issues. For the experimental studies, a lack of information reported in the papers meant that many study attributes were rated as ‘unclear’, most likely due to reporting restrictions in the respective journals. Finally, for one of the qualitative studies, information to demonstrate the rigour of the data analysis was not provided. All quantitative studies employed a methodological approach appropriate to the research topic and most undertook appropriate analyses, with the remaining four being unclear as to exactly how they analysed the data. However, for the majority of the studies it was not possible to determine whether a representative sample and objective measures (e.g. validated survey questions) had been used, with only studies, typically the experimental ones, using quota sampling to ensure samples were representative. Less than half of the studies justified their sample size or reported the response rate during recruitment. Finally, in terms of the quality of the papers, it may be that key methodological issues were not reported, rather than these being weak areas of study design, although this is potentially interpretable as evidence of bias. In the absence of validated quality appraisal tools, a best match was used.

**Table 3:** Quality appraisal of qualitative papers

Study	Was there a clear statement of aims?	Is a qualitative methodology appropriate?	Was the research design appropriate to the aims?	Was the recruitment strategy appropriate to the aims?	Were the data collected in a way that addressed the research issue?	Has the relationship between researcher and participant been adequately considered?	Have ethical issues been taken into consideration?	Was the data analysis sufficiently rigorous?	Is there a clear statement of findings?
Interviews									
Becker (2013)	Yes	Yes	Unclear	Yes	Yes	No	Unclear	Yes	Yes
Gupta et al. (2015)	Yes	Yes	Yes	Yes	Yes	Unclear	Unclear	Yes	Yes
Gupta et al. (2012)	Yes	Yes	Yes	Yes	Yes	Unclear	Unclear	Yes	Yes
Köhler and Som (2008)	Yes	Yes	Yes	Yes	Yes	No	Unclear	Unclear	Yes
Focus groups									
Brown et al. (2015)	Yes	Yes	Yes	Yes	Yes	Unclear	Unclear	Yes	Yes
Brown and Kuzma (2013)	Yes	Yes	Yes	Yes	Yes	Unclear	Unclear	Yes	Yes
Mixed methods									
Handford et al. (2015)	Yes	Yes	Yes	Yes	Yes	Unclear	Yes	Yes	Yes
Simons et al. (2009)	Yes	Yes	Unclear	Unclear	Unclear	Unclear	Unclear	Unclear	Yes
Yawson and Kuzma (2010)	Yes	Yes	Unclear	Unclear	Unclear	Unclear	Yes	Unclear	Unclear

**Table 4:** Quality appraisal of quantitative studies

Study	Was a survey appropriate for the aim?	What was the response rate?	Is the sample representative of the population?	Are the measures reported objective and reliable?	Was there a justification of the sample size?	Were appropriate statistical analyses performed?	Was there evidence of any other bias?
Surveys							
Arnold (2014)	Yes	Unclear	Unclear	Unclear	No	Unclear	Unclear
Besley et al. (2008)	Yes	32.3 %	No	Unclear	No	Yes	Yes
Capon et al. (2015)	Yes	19–48 %	Unclear	Yes	Yes	Yes	Yes
Cobb and Macoubrie (2004)	Yes	38–48 %	Unclear	Unclear	Unclear	Yes	No
Conti et al. (2011)	Yes	51.9 %	Unclear	Unclear	Unclear	Unclear	Unclear
Cook and Fairweather (2007)	Yes	29.6 %	No	Yes	No	Yes	No
Farshchi et al. (2011)	Yes	Unclear	Yes	Yes	Unclear	Yes	Unclear
Groves (2013)	Yes	71 %	No	Unclear	No	Yes	Unclear
Gupta et al. (2013)	Yes	32 %	Unclear	Unclear	No	Yes	Unclear
Schnettler et al. (2013a, b)	Yes	Unclear	Unclear	Unclear	Yes	Yes	Unclear
Schnettler et al. (2013a, b) neophobia	Yes	68 %	No	Yes	Yes	Yes	Yes
Schnettler et al. (2014)	Yes	Unclear	No	Yes	Yes	Yes	Yes
Siegrist et al. (2007)	Yes	Unclear	No	Unclear	No	Yes	Unclear
Siegrist et al. (2008)	Yes	28 %	Unclear	Unclear	No	Yes	Unclear
Siegrist et al. (2009)	Yes	43 %	Unclear	Yes	No	Yes	Yes
Stampfli et al. (2010)	Yes	41 %	Unclear	Unclear	Unclear	Yes	No
Suhaimee et al. (2014)	Yes	Unclear	Unclear	Unclear	No	Yes	Yes
Yue et al. (2015a, b)	Yes	86 %	No	Yes	No	Yes	Unclear
Experiments							
Bieberstein et al. (2013)	Yes	Unclear	Yes	Unclear	Unclear	Yes	No
Marette et al. (2009)	Yes	Unclear	Yes	Unclear	Unclear	Yes	No
Roosen et al. (2011)	Yes	Unclear	Yes	Unclear	Unclear	Yes	No
Conjoint analysis							
Casolani et al. (2015)	Yes	Unclear	Yes	Yes	No	Yes	Yes
Yue et al. (2015a, b)	Yes	97.5 %	Yes	Yes	No	Yes	Unclear
Mixed methods							
Handford et al. (2015)	Yes	8.67 %	Yes	Yes	Yes	Yes	Unclear
Roosen et al. (2015)	Yes	Unclear	Unclear	Yes	No	Yes	Unclear
Simons et al. (2009)	Yes	Unclear	Yes	Unclear	No	Unclear	Unclear
Yawson and Kuzma (2010)	Yes	30 %	Unclear	Unclear	No	Unclear	Unclear

The results below present the main themes that were identified from the thematic analysis (see Table 5). We indicate the relevant supplementary data boxes which are pertinent to each theme throughout the next section.

**Table 5:** Analytical themes

Theme 1	Type and applications of food nanotechnology
Theme 2	Benefits and risks of food nanotechnology
Theme 3	Socio-demographic influences
Theme 4	Creating an informed and trusting consumer
Theme 5	Characteristics of food nanotechnology
Theme 6	Link to historical agri-food technology concerns
Theme 7	Marketing and commercialisation
Theme 8	Future applications of agri-food nanotechnology

### Theme 1: type and applications of agri-food nanotechnology

Nanotechnology can be integrated into food products, can form part of the packaging of food, and/or can be used when processing food products. When considering these three types of application, overall, the majority of the studies (regardless of sample population) reported greater consumer acceptance of nanotechnology when it was applied to agri-food packaging and processing activities, compared to when it was integrated into agri-food products (see Supplementary Box 1).

Both consumer and expert opinion were divided on whether they found nanotechnology to be acceptable or unacceptable when used directly in foods as such. Experts appear to rate nanotechnology when applied to food and food products to be more acceptable than do consumers, but that could be because many of these experts worked in the nanotechnology field hold some asymmetric information (i.e. greater knowledge and information about risk and benefit assessment which is not available to consumers).

### Theme 2: benefits and risks of agri-food nanotechnology

Often agri-food-related nanotechnology was considered acceptable by experts when clear benefits could be identified. Experts considered benefits in relation to food freshness and safety, and wider environmental and food manufacturing advantages. In particular, if nanotechnology could prevent food spoilage and enhance the shelf-life of the food, and reduce the amount of packaging that would need to be used, it was viewed as acceptable. Additional wider applications of nanotechnology included using nanotechnologies to reduce food shortages, and to improve (reduce) calorie content of food. Ultimately, if the perceived benefits were thought to outweigh the perceived risks then nanotechnology applied to agri-food production was acceptable (see Supplementary Box 2a).

The available evidence suggests that consumers view agri-food nanotechnology favourably, for example in comparison to other agri-food technology innovations recently introduced such as genetically modified (GM) foods. Moreover, if the technology results in cheaper consumer products, and when it could assist beneficial food modifications (such as improved taste and disease prevention), it was perceived as acceptable. As found in the expert studies, the consumer studies found that if the perceived benefits outweighed the perceived risks, then agri-food nanotechnology is more acceptable to consumers (see Supplementary Box 2b).

The ‘commercializers’ perceived agri-food nanotechnology to be societally acceptable, although this may be attributable to participant’s professional roles in promoting such products (see Supplementary Box 2c). Ultimately, commercialisers viewed agri-food nanotechnology to be novel, to pose a low risk to individuals in terms of health impacts, and to be societally acceptable given that there are “riskier” technologies within the marketplace (although it was not clear to which ‘riskier’ technologies participants were referring in the published research).

However, both experts and consumers expressed concerns about the potential risks associated with using nanotechnology to produce food and food products. Experts perceived a greater risk associated with nanotechnology applied to the production of food products directly as compared to food packaging (see Supplementary Box 2d).

Experts and commercialisers noted that, even when nanotechnology was used in food packaging, there may be the potential for it to contaminate food with which it came into contact, increasing risks to consumers (see Supplementary Box 2e). The proximity of nanoparticles to the human body, and in particular ingestion of the particles, was viewed as high risk, and hence unacceptable by some experts.

Within the consumer studies, multiple concerns were raised. These included concerns about potential side effects, and beliefs that the technology could be misused; both of these concerns were underpinned by a fear of the unknown (see Supplementary Box 2f). Agri-food nanotechnology was also considered to be unacceptable because foods containing the technology are not perceived to be “natural” products. There was also a concern that nanotechnology is used for increasing profit, rather than for producing improved food products with discrete consumer benefits.

### Theme 3: socio-demographic influences

The studies included in the review are heterogeneous in nature and so it is difficult to conclusively link opinions about agri-food nanotechnology to individual socio-demographic characteristics. However, there is some indication that certain population groups may be more accepting of agri-food nanotechnology than others (see Supplementary Box 3). In particular, white, male population groups perceive fewer risks to be associated with the application of nanotechnologies. In terms of expert opinion regarding perceived acceptance, Europeans and Australasians appeared to be less open to agri-food nanotechnology than other population groups. In addition, those who are traditional in their outlook may perceive greater risks to the use of agri-food nanotechnologies, compared to those who are open to new technologies. However, in most of these studies no explanation was provided to explain how and why these particular socio-demographic groups may influence levels of consumer acceptance of agri-food nanotechnology.

### Theme 4: creating an informed and trusting consumer

The available evidence suggests that consumer acceptance of agri-food nanotechnologies may increase if there is clarity regarding who takes responsibility for creating and regulating safe nanotechnology products, as well as regarding who provides information about safety to the general public (see Supplementary Box 4a). Although regulations regarding the protection of human health is an obvious requirement for the effective commercialisation of any agri-food technologies, participants indicated that (harmonised) regulations are also required to facilitate trade of food products developed using nanotechnology across countries (see Supplementary Box 4b). Whether or not information should be provided through product labels, to inform consumers that particular products have been produced using nanotechnology, was a more contentious issue. It is unclear how much information consumers should be provided with, nor who should be responsible for educating and informing consumers about agri-food nanotechnology (see Supplementary Box 4c). Underpinning consumer acceptance (or rejection) of foods made using nanotechnology was the issue of trust. There is evidence that a higher level of trust in the nanotechnology industry was linked to greater acceptance of the technology (see Supplementary Box 4d). Consumers place a greater degree of trust in nanotechnology when it was used in food packaging compared to when it is integrated into food products.

Many studies indicated that consumers have limited knowledge about nanotechnology and how it can be applied to food products. For some consumers this may encourage early adoption of the technology, for others it can create concerns. Low levels of knowledge about nanotechnology may translate into a lower willingness to accept and purchase agri-food nanotechnology products because of a lack of understanding of how it is used in the food (see Supplementary Box 4e).

Commercialisers recognised that, in order to increase consumer acceptance of, and trust in, agri-food nanotechnology, rigorous testing of products may have to be undertaken by companies who use nanotechnology in their products (see Supplementary Box 4f). Being prepared for regulatory and labelling changes was deemed important, to help increase consumer confidence in agri-food nanotechnology, even if there was some scepticism about how well consumers would understand labelling of nanotechnology in agri-food products (see Supplementary Box 4g).

### Theme 5: characteristics of food nanotechnology

Acceptance of agri-food nanotechnology appears to be partly determined by the technology underpinning nanotechnology products, product characteristics and the cost of nanotechnology products (see Supplementary Box 5a). Those who preferred foods to be produced using “natural” processing methodologies, and who associated this with being healthy, perceived nanotechnology to be less acceptable, due to greater perceptions of risk. If agri-food nanotechnology brings tangible and concrete advantages to consumers (e.g. in relation to increased food security), then experts are more likely to rate the different applications as acceptable (see Supplementary Box 5b). Consumers were however, not willing to pay more for products developed using nanotechnology, independently of the benefits that will be delivered through its application.

### Theme 6: link to historical agri-food technology concerns

In some of the studies reviewed, consumers linked agri-food nanotechnology to GM foods. This may have lowered the acceptability of agri-food nanotechnology if GM foods are perceived negatively (see Supplementary Box 6). Where there was consumer uncertainty about the acceptability of agri-food nanotechnology, individuals utilised their existing “reference points” to assess the risks and benefits arising from the technology. As one of these reference points is potentially GM foods, this may have created lower consumer acceptance of agri-food nanotechnology.

### Theme 7: marketing and commercialisation

In order to encourage consumer purchases of agri-food nanotechnology products, the role of marketing and, in particular, branding is potentially an important topic of research. Highlighting the benefits to consumers via marketing communications was rated important, as was the development of a “trustworthy brand”. These recommendations are not dissimilar to the role marketing plays for other types of products and services (see Supplementary Box 7a).

It was recognised that encouraging increased repeat purchases of agri-food nanotechnology would inspire confidence in other population groups and thus increase acceptance. Thus it was suggested that those consumers who view agri-food nanotechnology to be most acceptable may “lead” in terms of technology adoption, which may then open up the market for other agri-food nanotechnology products (see Supplementary Box 7b). It was also reported that food packaging should be commercialised ahead of foods produced using nanotechnology, as this would be more acceptable to consumers. Furthermore, informed expert opinion might usefully be utilised to facilitate the formation of consumer opinions regarding agri-food nanotechnology and its potential acceptability by consumers.

### Theme 8: future applications of agri-food nanotechnology

Most recommendations for future research focused on understanding the determinants of consumer acceptance of food nanotechnology in different cultures. Comparing expert and consumer opinion was considered an important research area, as there may be a mismatch between what experts would provide in terms of agri-food nanotechnology and what would be accepted by consumers (see Supplementary Box 8a). This applied to future developments as well as those currently well advanced in terms of their innovation trajectories.

When consumer characteristics were considered in the studies reviewed, there was a focus on demographic characteristics rather than wider psychographic characteristics. Thus, moving beyond the focus on socio-demographic characteristics and to consider other psychological and cultural determinants was also identified as important (see Supplementary Box 8b). For example, consumers with an internal “health locus of control” (who perceive that they are able to influence their own health status through their behaviours) may be more inclined to adopt consumer products with distinct health benefits (Poínhos et al. 2014).

Exploring the drivers of social negativity towards new technologies, as well as risk aversion in the context of agri-food nanotechnology, were identified as future research priorities (see Supplementary Box 8c). Furthermore, there was a call for consumer acceptance research to use real nanotechnology products, rather than hypothetical scenarios, in order to provide study participants with a real experience of such products. This could help to provide a more realistic evidence base regarding consumer acceptance of nanotechnology, although it is clearly dependent on both the product innovation trajectory and regulatory approval of such products, in particular if they were consumed by study participants, or in some other way come into physical contact with consumers.

Finally, other key issues were identified that might influence consumer acceptance of agri-food nanotechnology. These considerations also related to the themes identified above, particularly providing clear and detailed information, involving multiple stakeholders in the debate on nanotechnology and building consumer confidence and trust (see Supplementary Box 8d).

## Discussion

### Statement of main findings

We believe that this is the first systematic review to explore empirical findings reporting on consumer and expert acceptance of nanotechnology applied to the agri-food sector. Included in this review are 32 empirical studies focused on consumer and expert opinions towards agri-food nanotechnology. The majority of these studies used a survey methodology to assess acceptance, although each survey asked very different questions of participants. In-depth empirical (i.e. qualitative research), or experimental research (for example, that which examined the impacts of information interventions on consumer attitudes) exploring consumer acceptance was limited, and it may be useful to follow this up in future research. The analysis of the research reported in the papers included in the review identified eight themes which appear relevant to understanding societal acceptance of agri-food nanotechnology. The consumer studies, and those involving expert assessment of consumer perceptions, suggested that the benefits and risks which consumers perceive to be associated with nanotechnology applied to food production and food products is likely to be an important determinant of consumer responses. In this respect, agri-food nanotechnology is likely to be accepted by consumers if the perceived benefits in some way outweigh the perceived risks and associated consumer concerns. In particular, nanotechnology was deemed more acceptable when it was used in food packaging and processing rather than as an integral part of food products themselves. It was also found that agri-food nanotechnology may be more acceptable if it results in cheaper, safer, consumer products, i.e., a tangible and desirable consumer benefit is delivered as a consequence of its application.

There is reasonable consistency in the literature regarding societal acceptance of agri-food applications of nanotechnology. Although consumers express some concerns about nanotechnology applied to food production *per se*, less concern is expressed about nanotechnology applied to innovative novel food packaging. However, the consumer rejection of nanotechnology applied to food production, anticipated by some stakeholders, and following consumer reaction to GM applied to food production in some parts of the world, has not been supported by the evidence identified in this review. Increased inputs by consumers into the product development process, when concrete and tangible consumer benefits are being incorporated into specific products, is required to ensure what is being developed is also what consumers want (Raley et al. In Press).

Our systematic review has also highlighted a major gap in the available literature which concerns research which utilises theoretical approaches to understanding societal acceptance of nanotechnology applied to agri-food production. Developing research which is theoretically informed is potentially advantageous insomuch as it may facilitate greater ability to predict consumer’s requirements of nanotechnological innovation in the future. Utilising theoretically driven approaches will also enable more systematic comparison of research outcomes across studies (for example, between populations with different characteristics, with respect to societal acceptance of different applications, and analysis of trends on consumer acceptance with time), in particular if a common theoretical or methodological framework or approach is adopted. It is also notable that many of the studies included in the review identified further exploration of the drivers of social negativity towards new technologies, as well as social negativity and risk aversion as future research priorities. Given that one conclusion of this systematic review is that perceived benefit is a relevant and important determinant of consumer behaviour, it will also be important to understand drivers of acceptance and benefit acquisition. It would be useful if future research systematically integrated both risk and benefit perception analyses in the research design, not least because benefit information might usefully be applied to refining the product development trajectory in the future. Commercial success will depend on consumers perceiving tangible and concrete benefits to be associated with the application of nanotechnology to food products.

### Strength and weaknesses of studies included in the review

The majority of the studies reviewed used quantitative survey methodologies. Often large—and sometimes nationally representative—samples were used. This facilitated comparative analysis of the acceptance of agri-food nanotechnology across different consumer segments but did not allow for exploration or in-depth analysis of why these views were held by consumers, given the method used to collect the data. Three studies utilised experimental methodologies (i.e. choice experiments) to explore consumer preferences for (hypothetical) food nanotechnology products. Consumer experience (whether positive or negative) of foods produced using nanotechnology may influence subsequent choice behaviours, and as such limit the generalisability of findings from studies using choice experiments.

In addition, the application of formal quality appraisal indicated that studies were poor at reporting sampling and analytical procedures, and often ethical approvals for research which utilised human participants. However, the studies assessed acceptance of agri-food nanotechnology across a wide range of stakeholders, including representative groups of consumers, experts and commercialisers, as well as reporting data from a cross-section of participants, from multiple countries and backgrounds. Therefore, whilst the findings of this review highlight acceptance of agri-food nanotechnologies from the perspective of multiple stakeholders, further research is required to see how the gap can be narrowed between expert/commercialiser opinions and consumer views, to ensure nanotechnologies are acceptable to consumers, whilst being commercially viable to those who produce such technologies.

### Strengths and weaknesses of this review

We believe that this systematic review has captured the available empirical evidence exploring consumer and expert opinion towards agri-food nanotechnology. Similar findings are reported across the included papers, and so we are confident that we have reached data saturation (Francis et al. 2009) regarding consumer and expert acceptance of agri-food nanotechnology. In particular, this systematic review affords those interested in commercialising nanotechnology with a quick reference guide to consumer and expert opinions towards nanotechnologies when applied to agri-food products and production methods. This review synthesises the factors that both help and hinder food nanotechnology commercialisation and provides suggestions for future research, legislation of nanotechnology and consumer education. By synthesising all of the relevant literature in these areas, this systematic review allows those interested in the field to gain an oversight of these key issues much more quickly than would occur by reading individual papers. Aggregation of the literature in this systematic review allows readers an opportunity to identify key issues, areas of concern and future developments in the field that would not be obtainable by reading individual papers in a stand-alone context.

Whilst the authors are of the opinion that data saturation was reached, 17 papers were excluded because they were unobtainable in English and/or they were unavailable. Likewise, we have not reviewed the grey literature in this area, and so again, we may have missed relevant opinions that have not been published in English language peer-reviewed journals. Some of the papers refer to grey literature, such as the Eurobarometer (European Commission 2010), but they do not discuss themes that are wholly different to the results of our systematic review.

A further weakness is that we have been unable to undertake a quantitative meta-analysis given the heterogeneity of dependent variables across the included papers. However, it may be feasible to revisit this review at a future date to conduct a meta-analysis, once there are a greater number of published empirical studies in this area which report suitable data.

### Implications for policy and practice

A consistent finding was that acceptance depends on the perceived benefits of nanotechnology outweighing the perceived risks, although there is less consistency in reporting what constitutes a “desirable benefit” in terms of consumer perceptions. Benefits may refer to generic factors like (cheaper) prices or benefits specific to different agri-food applications. Systematic analysis of what these preferred benefits are, and which consumers want them, is needed. Policy makers and other stakeholders should also be aware that much of the research indicated that, for agri-food nanotechnology to be accepted in the marketplace, consumer confidence and trust in nanotechnology, food manufacturers, regulators and nanotechnology experts, must be developed and maintained. This might be achieved, for example, through good technology governance practice, e.g. (see Bernstein et al. 2014; Marchant 2012), effective risk–benefit communication, (Binder et al. 2011; Frewer et al. 2015) and stakeholder and end-user involvement on technology development, in line with best practice in responsible Research and Innovation policies (de Bakker et al. 2014; von Schomberg 2013).

A focus on communicating the potential benefits and risks of nanotechnology, building on consumer concerns, and investigation of how food nanotechnology can be regulated in a way that inspires consumer confidence, will increase the likelihood of food nanotechnology purchases.

## Conclusion

Nanotechnology is more likely to be accepted in food packaging rather than integrated into food products. Trust and confidence in agri-food nanotechnology needs to be fostered, to increase consumer acceptance. Providing information to consumers on the benefits of nanotechnology, and ensuring an informed public could help to reduce consumer concern and could inspire food nanotechnology purchases. However, research is needed to understand what consumers perceive as beneficial, as well as how they construe risks. Adopting theoretically underpinned approaches to understanding consumer perceptions and attitudes will facilitate comparative analysis across different groups of consumers, different food nanotechnology applications, and allow assessment of trends in consumer priorities and concerns with time.

## Electronic supplementary material

Supplementary material 1 (DOCX 84 kb)

## Footnotes

1 Prospero is a well-known database of systematic reviews in health and social issues, ran by the CRD. The CRD produces three databases: Database of Abstracts of Reviews of Effects; NHS Economic Evaluation Database; and Health Technology Assessment Database. Whilst these databases are not wholly applicable to our review, they are the only databases of their kind to register systematic reviews, and thus were checked for thoroughness. Any other systematic reviews on a similar topic would have been returned in our searches of the main databases for relevant papers.
