# Supplementary material for: Consumer acceptance of and willingness to pay for food nanotechnology: a systematic review
Source: J Nanopart Res. 2015 Nov 30;17:467. doi: 10.1007/s11051-015-3270-4 (PMC4666279; doi:10.1007/s11051-015-3270-4)
Supplement: Supplementary file 1 — Supplementary material 1 (DOCX 84 kb) [file 11051_2015_3270_MOESM1_ESM.docx]

**SUPPLEMENTARY FILES**

**Supplementary** **Data 1: Protocol**

**Supplementary** **Data 2: PRISMA checklist**

**Supplementary** **Data 3: Example search terms**

**Supplementary** **Data 4: Box 1 Quotations to illustrate the ‘Type and applications of food nanotechnology’ theme**

**Supplementary** **Data 5: Box 2 Quotations to illustrate the ‘Benefits and risks of agri-food nanotechnology’ theme**

**Supplementary** **Data 6: Box 3 Quotations to illustrate the ‘Socio-demographic influences’ theme**

**Supplementary** **Data 7: Box 4 Quotations to illustrate the ‘Creating an informed and trusting consumer’ theme**

**Supplementary** **Data 8: Box 5 Quotations to illustrate the ‘Characteristics of agri-food nanotechnology’ theme**

**Supplementary** **Data 9: Box 6 Quotations to illustrate the ‘Link to historical agri-food nanotechnology concerns’ theme**

**Supplementary** **Data 10: Box 7 Quotations to illustrate the ‘Marketing and commercialisation’ theme**

**Supplementary** **Data 11: Box 8 Quotations to illustrate the ‘Future applications of food nanotechnology’ theme**

**Supplementary** **Data 1: Protocol**

**Protocol**

*SAFRD, Newcastle University*

27 November 2014

**1. REVIEW TITLE**

*Review title*

How acceptable is nanotechnology, when applied to food and food products, to consumers?

**2. REVIEW TEAM CONTACT DETAILS**

*Named contact & organisational affiliation of the review*

Named contact:

[removed for peer review]

*Review team members & organisational affiliations*

[removed for peer review]

*Funding sources/sponsors*

N/A.

*Conflicts of interest*

None known

*Collaborators*

Not applicable

**3. REVIEW METHODS**

*Primary research question*

How acceptable is nanotechnology to consumers, when applied to food and food products?

*Additional research questions*

1. What are consumer attitudes towards nanotechnology?

2. What are consumer and expert attitudes/perceptions towards nanotechnology when applied to food and food production? Including:

a. Beliefs

b. Values

c. Risks/Benefits

d. Concerns

3. What is the influence of consumer attitudes and perceptions on their intention to consume and purchase food-related nanotechnology applications?

*Condition or domain being studied & context*

Nanotechnology utilises scientific advancements in the study of “molecules, compounds, or particles at the extremely small scale of about a millionth of a millimetre” (Cook and Fairweather, 2007). Its uses can vary; including in cosmetics, medicine, electronics, IT, textiles, and for environmental solutions, military use and space exploration (Economic and Social Research Council [ESRC], 2003). In particular relation to food, food production and food packaging, nanotechnology can be applied in the processing of commodities, such as in flour milling, or in functional foods whereby bioactive compounds are added to foods to create foods with additional physiological benefits (Sozer and Kokini, 2008). Nanoparticles can also be used in food packaging, to make packaging that is biodegradable and more environmentally friendly (Sozer and Kokini, 2008).

That said, nanotechnology in food products, processes and packaging presents numerous safety concerns, as well as “environmental, ethical, policy and regulatory issues” (House of Lords, 2010: 1). Whilst there are toxicological tests which are available to monitor the risk of nanotechnology in food, there are still concerns that the ‘standard’ tests are unable to detect very small effects (House of Lords, 2010). Due to such safety (amongst other) concerns, food consumers are often sceptical of nanotechnology in food (ESRC, 2003; Siegrist et al, 2009; Frewer, 2003). Whilst the picture is mixed, studies have found that consumers are unwilling to accept nanotechnology in foods, even if the health benefits are obvious, although there is a greater acceptance towards nanotechnology in food packaging (Siegrist, 2009). It is argued that greater public engagement with food nanotechnology may help to ease consumer concerns around its use, but that limited research has to-date been undertaken that can link risk assessment, consumer concerns, public engagement and nanotechnology in the food arena (Kuzma et al, 2008).

Consumer acceptance of nanotechology in food is important, considering that it can help to combat pressing global concerns, such as food shortages (ESRC, 2003). That said, whilst there has been some attempt to conduct systematic reviews of the regulatory situation surrounding nanotechnology (Grobe et al, 2008), systematic reviews exploring consumer attitudes, perceptions and acceptance of nanotechnology in relation to food is less common. Searching the PROSPERO database - a database containing registered systematic reviews in health and social care (PROSPERO, 2012) - and the databases of the Centre for Reviews and Dissemination (Centre for Reviews and Dissemination, 2012) does not indicate directly applicable systematic reviews in the areas of consumer acceptance, expert opinion, food and nanotechnology (Besley et al, 2008). The systematic reviews that have been conducted are in the general area of nanotechnology or focus on specific food issues, such as vitamin D food fortification (Black et al, 2011). Thus, it can be suggested that this area is under-researched.

This research seeks to provide policy makers, nanotechnology experts, and food manufacturers with a systematic review of the evidence concerning societal acceptance of nanotechnology and food. By undertaking this systematic review, we will offer policy makers and industry with all of the available evidence surrounding consumer acceptance of food nanotechnology. This will assist them in their decision making, risk assessment approaches, and will be prudent since they will have an indication of how consumers may react to future products, rather than waiting for the ‘aftermath’ to occur after food nanotechnology products are released (Cook and Fairweather, 2007).

*Overview of the search strategy*

Research reports for inclusion in the review will primarily be found through database searches, using search engines. There will be no systematic hand searching of journals or conference proceedings.

*Inclusion criteria*

Peer-reviewed papers will be included in the review if they meet all of the following criteria:

 Language: English.

 Date range: All dates.

 Study design: Empirical study including both qualitative and quantitative data.

 Population: Adults (aged 18 years or over).

 Intervention: Must contain a discussion of nanotechnology in relation to agri-food, risk perceptions, consumer acceptance, policy implications and research applications.

 Outcome measure: Discussion of stakeholder attitudes towards nanotechnology applied to food and food production.

*Search strategies*

Peer reviewed literature will be included in the systematic review. The following sources will be searched to identify published literature:

 Electronic databases of peer-reviewed journal articles; Scopus, Web of Knowledge, CAB Abstracts, PsychInfo, Medline, and Embase.

 The reference lists of all studies that meet the inclusion criteria, as well as relevant reviews will be scanned to identify further relevant publications.

The search strategy will take the general form of: nanotechnology AND terms for consumer acceptance, risk, and agri-food, and will be developed with the help of a specialist librarian. The search term will be adapted for use in each electronic medium.

*Screening*

After importing search results into EndNote and removing duplicates, screening will be conducted in three independent phases. Firstly, titles will be screened by two researchers (ELG and BC) independently to identify publications that do not meet the inclusion criteria. These publications will then be excluded with brief notes taken on the reasons for their exclusion. In cases of doubt, publications will be included for further discussion.

Secondly, the abstracts of publications that were included in the first screening round will be screened again by the same two researchers, to identify those that definitely do not meet the inclusion criteria. In any cases of doubt, or where an abstract is not present, publications will be included. Reasons for exclusion will again be noted.

Finally, the full text of publications that were included following the second screening will be screened by the same two researchers. On this occasion the assessment will be whether publications meet the inclusion criteria, with notes made on whether they meet all of the criteria. Any disagreements at this stage will be resolved by discussion. Only papers that meet all of the inclusion criteria will be kept, with tables of excluded studies prepared, detailing when exclusion occurred and reasons for exclusion.

*Primary outcome(s)*

Debate on consumer acceptance of nanotechnology as it is applied to food and food production.

*Secondary outcome(s)*

We include here all additional variables of interest: risk perceptions of food and nanotechnology, political discussion on food and nanotechnology, and research applications in the area of food and nanotechnology.

*Data extraction (selection and coding)*

A coding framework will be developed using Nvivo software, and will include: participant characteristics, the research method, year of data collection, sample size and method, location of research data collection, and quality assessment. Data will be extracted by one reviewer and checked by a further two reviewers. Any disagreements will be resolved by discussion.

Where publications lack details required for quality assessment or full data extraction, authors will be contacted to request further details.

*Risk of bias/quality assessment*

The quality of all studies that meet the inclusion criteria will be formally assessed and will be assessed by researchers working independently using the Petticrew and Roberts and CASP tools for quantitative and qualitative data.

*Strategy for data synthesis and reporting*

We will begin by describing the range of debate in the area (both consumer and expert opinions), the theoretical and empirical rationales used to guide the debate in the area, population characteristics, and the political and research outcomes that have been studied. Finally, we will prepare a Table of Included Studies.

**4. GENERAL INFORMATION**

*Type of review*

Systematic review with possible meta-analysis.

*Language*

English

*Country*

United Kingdom

*Dissemination plans*

In order to disseminate our findings to the academic community, we will write up and submit our results for publication in a peer-review journal (e.g. Nature Nanotechnology).

*Keywords*

Systematic review, nanotechnology, consumer acceptance, risk perceptions, agri-food, food and food production.

*Details of any existing review of the same topic by the same authors*

None.

**Supplementary** **Data 2: PRISMA checklist**

| **Section/topic** | **#** | **Checklist item** | **Reported on page #** |
| --- | --- | --- | --- |
| **TITLE** | | |  |
| Title | 1 | Identify the report as a systematic review, meta-analysis, or both. | 1 |
| **ABSTRACT** | | |  |
| Structured summary | 2 | Provide a structured summary including, as applicable: background; objectives; data sources; study eligibility criteria, participants, and interventions; study appraisal and synthesis methods; results; limitations; conclusions and implications of key findings; systematic review registration number. | 2-3 |
| **INTRODUCTION** | | |  |
| Rationale | 3 | Describe the rationale for the review in the context of what is already known. | 4-5 |
| Objectives | 4 | Provide an explicit statement of questions being addressed with reference to participants, interventions, comparisons, outcomes, and study design (PICOS). | 5-7 |
| **METHODS** | | |  |
| Protocol and registration | 5 | Indicate if a review protocol exists, if and where it can be accessed (e.g., Web address), and, if available, provide registration information including registration number. | 5 |
| Eligibility criteria | 6 | Specify study characteristics (e.g., PICOS, length of follow-up) and report characteristics (e.g., years considered, language, publication status) used as criteria for eligibility, giving rationale. | 6 |
| Information sources | 7 | Describe all information sources (e.g., databases with dates of coverage, contact with study authors to identify additional studies) in the search and date last searched. | 5-6 |
| Search | 8 | Present full electronic search strategy for at least one database, including any limits used, such that it could be repeated. | Supp. Data 3 |
| Study selection | 9 | State the process for selecting studies (i.e., screening, eligibility, included in systematic review, and, if applicable, included in the meta-analysis). | 6-7 |
| Data collection process | 10 | Describe method of data extraction from reports (e.g., piloted forms, independently, in duplicate) and any processes for obtaining and confirming data from investigators. | 6-7 |
| Data items | 11 | List and define all variables for which data were sought (e.g., PICOS, funding sources) and any assumptions and simplifications made. | 6-7 |
| Risk of bias in individual studies | 12 | Describe methods used for assessing risk of bias of individual studies (including specification of whether this was done at the study or outcome level), and how this information is to be used in any data synthesis. | 6-7 |
| Summary measures | 13 | State the principal summary measures (e.g., risk ratio, difference in means). | 5-7 |
| Synthesis of results | 14 | Describe the methods of handling data and combining results of studies, if done, including measures of consistency (e.g., I^2^) for each meta-analysis. | 5-7 |
| Risk of bias across studies | 15 | Specify any assessment of risk of bias that may affect the cumulative evidence (e.g., publication bias, selective reporting within studies). | 5-7 |
| Additional analyses | 16 | Describe methods of additional analyses (e.g., sensitivity or subgroup analyses, meta-regression), if done, indicating which were pre-specified. | n/a |
| **RESULTS** | | |  |
| Study selection | 17 | Give numbers of studies screened, assessed for eligibility, and included in the review, with reasons for exclusions at each stage, ideally with a flow diagram. | 7-8 |
| Study characteristics | 18 | For each study, present characteristics for which data were extracted (e.g., study size, PICOS, follow-up period) and provide the citations. | 7-8 |
| Risk of bias within studies | 19 | Present data on risk of bias of each study and, if available, any outcome level assessment (see item 12). | 8-9 |
| Results of individual studies | 20 | For all outcomes considered (benefits or harms), present, for each study: (a) simple summary data for each intervention group (b) effect estimates and confidence intervals, ideally with a forest plot. | n/a |
| Synthesis of results | 21 | Present results of each meta-analysis done, including confidence intervals and measures of consistency. | n/a |
| Risk of bias across studies | 22 | Present results of any assessment of risk of bias across studies (see Item 15). | 8-9 |
| Additional analysis | 23 | Give results of additional analyses, if done (e.g., sensitivity or subgroup analyses, meta-regression [see Item 16]). | n/a |
| **DISCUSSION** | | |  |
| Summary of evidence | 24 | Summarize the main findings including the strength of evidence for each main outcome; consider their relevance to key groups (e.g., healthcare providers, users, and policy makers). | 14-16 |
| Limitations | 25 | Discuss limitations at study and outcome level (e.g., risk of bias), and at review-level (e.g., incomplete retrieval of identified research, reporting bias). | 16-17 |
| Conclusions | 26 | Provide a general interpretation of the results in the context of other evidence, and implications for future research. | 18 |
| **FUNDING** | | |  |
| Funding | 27 | Describe sources of funding for the systematic review and other support (e.g., supply of data); role of funders for the systematic review. | 1 |

**Supplementary** **Data 3: Example search terms**

TI=(consumer OR lay OR public OR customer OR expert OR stakeholder OR citizen OR people OR individual OR consumer attitude OR consumer behaviour OR consumer information OR consumer panel) AND (nano OR nanotechnology OR "nano material" OR nano products) AND (food OR food product OR product OR consumption OR purchase OR preparation OR storage)

TI=(accept* OR perception OR thought OR view OR belief OR factor OR idea) AND (society OR public OR group) AND (nano OR nanotechnology OR nanomaterial OR nano products) AND (food OR food product OR food production OR health) AND (consumer OR lay OR public OR customer OR expert OR stakeholder OR citizen OR people OR individual OR consumer attitude OR consumer behaviour OR consumer behavior OR consumer information OR consumer panel)

TI=( accept* OR perception OR thought OR view OR belief OR factor OR idea) AND (nano OR nanotechnology OR nanomaterial OR nano products) AND (consumer OR lay OR society OR public OR group OR customer OR expert OR stakeholder OR citizen OR people OR individual OR consumer attitude OR consumer behaviour OR consumer behavior OR consumer information OR consumer panel)

TI=(attitude OR value OR anxiety OR risk OR benefit OR concern OR impact OR accept* OR perception OR thought OR view OR belief OR factor OR idea) AND (nano OR nanotechnology OR nanomaterial OR nano products) AND (consumer OR society OR public OR group OR public OR customer OR lay OR expert OR stakeholder OR citizen OR people OR individual OR consumer attitude OR consumer behaviour OR consumer behavior OR consumer information OR consumer panel) AND (food OR food product OR food production OR health)

TI=(attitude OR value OR anxiety OR risk OR benefit OR concern OR impact OR accept* OR perception OR thought OR view OR belief OR factor OR idea) AND (nano OR nanotechnology OR nanomaterial OR nano products) AND (consumer OR lay OR public OR customer OR expert OR stakeholder OR citizen OR people OR individual OR consumer attitude OR consumer behaviour OR consumer behavior OR consumer information OR consumer panel OR society OR public OR group) AND (food OR food product OR food production OR health) AND (buy OR purchase)

TI=(accept* OR perception OR thought OR view OR belief OR factor OR idea) AND (nano OR nanotechnology OR nanomaterial OR nano products) AND (food OR food product OR food production OR health) AND (consumer OR lay OR public OR customer OR expert OR stakeholder OR citizen OR people OR individual OR consumer attitude OR consumer behaviour OR consumer behavior OR consumer information OR consumer panel OR society OR public OR group)

TI=(accept* OR perception OR thought OR view OR belief OR factor OR idea) AND (nano OR nanotechnology OR nanomaterial OR nano products OR technology OR engineering OR modified) AND (consumer OR public OR customer OR lay OR expert OR stakeholder OR citizen OR people OR individual OR consumer attitude OR consumer behaviour OR consumer behavior OR consumer information OR consumer panel OR society OR public OR group)

TI=(consumer OR public OR customer OR lay OR expert OR stakeholder OR citizen OR people OR individual OR consumer attitude OR consumer behaviour OR consumer behavior OR consumer information OR consumer panel) AND (nano OR nanotechnology OR "nano material" OR nano products OR technology OR engineering OR modified) AND (food OR food product OR product OR consumption OR purchase OR preparation OR storage)

TI=(accept* OR perception OR thought OR view OR belief OR factor OR idea) AND (nano OR nanotechnology OR nanomaterial OR nano products OR technology OR engineering OR modified) AND (food OR food product OR food production OR health) AND (society OR public OR group OR consumer OR public OR customer OR lay OR expert OR stakeholder OR citizen OR people OR individual OR consumer attitude OR consumer behaviour OR consumer behavior OR consumer information OR consumer panel)

TI=(attitude OR value OR anxiety OR risk OR benefit OR concern OR impact OR accept* OR perception OR thought OR view OR belief OR factor OR idea) AND (nano OR nanotechnology OR nanomaterial OR nano products OR technology OR engineering OR modified) AND (society OR public OR group OR consumer OR lay OR public OR customer OR expert OR stakeholder OR citizen OR people OR individual OR consumer attitude OR consumer behaviour OR consumer behavior OR consumer information OR consumer panel) AND (food OR food product OR food production OR health)

TI=(attitude OR value OR anxiety OR risk OR benefit OR concern OR impact OR accept* OR perception OR thought OR view OR belief OR factor OR idea) AND (nano OR nanotechnology OR nanomaterial OR nano products OR technology OR engineering OR modified) AND (society OR public OR group OR consumer OR lay OR public OR customer OR expert OR stakeholder OR citizen OR people OR individual OR consumer attitude OR consumer behaviour OR consumer behavior OR consumer information OR consumer panel) AND (food OR food product OR food production OR health) AND (buy OR purchase)

**Supplementary** **Data 4: Box 1 Quotations to illustrate the ‘Type and applications of agri-food nanotechnology’ theme**

| **Box 1:**  *“Participants were more willing to use nanotechnology food applications involving packaging…than either food additives...or processing” (*[*Brown and Kuzma 2013*](#_ENREF_11)*)* |
| --- |

**Supplementary** **Data 5: Box 2 Quotations to illustrate the ‘Benefits and risks of agri-food nanotechnology’ theme**

| **Box 2a:**  *“The use of nanoclay polymer-composites in food packaging would better protect food freshness, delay spoilage, and enhance the shelf life of packaged foods.”* ([Köhler and Som 2008](#_ENREF_35))  *“The scientists surveyed generally rate the risks of nanotechnology substantially lower than the beneﬁts.”* ([Besley et al. 2008](#_ENREF_5))  *“… with some aspect of addressing starvation, food supply, or food quality, with the top three sub- themes emerging as ‘‘Food preservation, spoilage prevention, and storage’’ … ‘‘Food distribution and production’’ … and ‘‘Better/enhanced nutrition or crop yields’’…” (Brown et al. 2015)*  *“…nanotechnology that reduced calorie content …” (Casolani et al. 2015)*  *“The complexity of participant views is illustrated by this participant’s comment: the focus was on using technology to adjust food production methods, in order to expand general food production and improve nutrition, while preserving the ability of the environment to support food production and ensuring that the beneﬁts go to not only the very rich.” (Brown et al. 2015)*  **Box 2b:**  *“Our data suggest that from Iranians’ view, the …* [largest] *beneﬁt of nanotechnology to achieve is new ways to detect and treat human diseases and the second high scored beneﬁt is cheaper, longer lasting consumer products.’’* ([Farshchi et al. 2011](#_ENREF_22))  *“Descriptive analysis showed that most of the people agree that nanotechnology is beneficial to them as it could modify foods based on nutritional needs or tastes.”* ([Suhaimee et al. 2014](#_ENREF_75))  **Box 2c:**  *“Interviewees responded that some nanomaterials and nanotechnologies were novel, and some were not. But overall, there was an insistence from subjects that nanotechnology has ‘been around forever’, and that what is new is our more complete understanding and control of matter at this small scale.”* ([Becker 2013](#_ENREF_3))  *“For these subjects, this was the case because of their belief that either (1) the small volume of production and exposure to nanoproducts made them less risky, (2) all individual nanomaterials agglomerate before coming into contact with humans, (3) nanotechnology is relatively less risky than other technologies currently on the market, such as genetically-modiﬁed organisms (GMOs) and organics, (4) nanotechnology’s risks are comparable to ultra-ﬁne particles (UFPs), (5) or that most nanomaterials on the market have been embedded within matrices so as to limit consumer exposure.”*([Becker 2013](#_ENREF_3))  *“Some emphasized the normalcy of risks accompanying newly developed technologies.”* ([Becker 2013](#_ENREF_3))  **Box 2d:**  *“People don’t think about nanoparticles when it is in their [tennis] rackets and sports equipment, but they start to think of risks if these particles are in food.”* ([Gupta et al. 2012](#_ENREF_30))  **Box 2e:**  *“Others pointed out that nanoparticles could potentially migrate from the packaging into the food and then pose a health risk.”* ([Köhler and Som 2008](#_ENREF_35))  *“Some subjects mentioned that, because of their small size, some nanomaterials are able to be taken up by cells and absorbed through the skin and that this presents a health risk.”* ([Becker 2013](#_ENREF_3))  **Box 2f:**  *“When it comes to food, in particular, the overwhelming majority of the population is against nanotechnology. Therefore, it is obvious that nanotechnology and food makes the majority feel at least uncomfortable and that it does not enjoy acceptance.”* ([Simons et al. 2009](#_ENREF_71))  *“In the context of food, nanotechnology is not natural, and hence, it goes against the common belief that natural is good and unnatural is bad.”* ([Simons et al. 2009](#_ENREF_71))  *“Finally, in terms of nanoenabled food, the robustness of bodily invasion in our experiments indicates that [nano]-food may trigger particularly strong reactions and concerns because it is consumed intentionally, but possibly unknowingly.”* ([Conti et al. 2011](#_ENREF_18))  *“…the main reasons for unwillingness to use nano-products were limited knowledge about the product and merely the fact that the product is new.” (Brown et al. 20115)* |
| --- |

**Supplementary** **Data 6: Box 3 Quotations to illustrate the ‘Socio-demographic influences’ theme**

| **Box 3:**  *“We ﬁnd that whites and more educated respondents are more likely to perceive beneﬁts exceeding risks.”* ([cobb and Macoubrie 2004](#_ENREF_16))  *“Consistent with the white male effect, white and male participants perceived the beneﬁts of nanotechnology as outweighing the risks as compared to women and non-whites.”* ([Conti et al. 2011](#_ENREF_18))  *“Men are signiﬁcantly more likely than women to think that beneﬁts outweigh risks. And individuals who have greater knowledge of nanotechnology are far more likely to say that the beneﬁts will outweigh the risks, and those who have no knowledge of the technology are more likely to say that the risks will outweigh the beneﬁts.”* ([Simons et al. 2009](#_ENREF_71))  *“Older respondents perceived nano-outside applications as signiﬁcantly more beneﬁcial than younger respondents. No signiﬁcant age effect was observed for nano-inside applications. Females perceived signiﬁcantly less beneﬁts associated with both nano- outside and nano-inside applications than males.”* ([Siegrist et al. 2008](#_ENREF_70))  *“Experts also indicated that agri-food applications of nanotechnology would be more acceptable in Northern America, Singapore and India and less so in Europe and Australasia.”* ([Gupta et al. 2013](#_ENREF_29))  *“The second segment … labelled “traditionalist displayed a strong negative utility for nano- technology produced wine” (Casolani et al. 2015)*  *“[Those] prone to nanotechnology… assigned greatest importance to the type of nanotechnology application in the food…” (Schnettler et al. 2014)* |
| --- |

**Supplementary** **Data 7: Box 4 Quotations to illustrate the ‘Creating an informed and trusting consumer’ theme**

| **Box 4a:**  *“Generally, responsibility for safe development was perceived as something shared by multiple parties. But there was a strong tendency for interviewees to emphasize their own company’s responsibility or industry’s responsibility for making safe products.”* ([Becker 2013](#_ENREF_3))  *“A couple of subjects indicated that consumers were under-protected because there was insufﬁcient knowledge about the safety of some nano-products entering the market.”* ([Becker 2013](#_ENREF_3))  **Box 4b:**  *“As might be expected, respondents see a need for regulation most clearly in those areas where they see the most risk, including issues related to human and animal health and protection of the natural environment…Health (human and animal), environmental, and privacy concerns were seen as the areas with the least adequate regulations, but not by a wide margin…With regard to regulations, it appears that many of the scientists involved see a need to appropriately manage potential risks. The priority for regulation seems to be in the areas of health and environmental regulation, with scientists also indicating that current regulations in these areas may not be adequate.”* ([Besley et al. 2008](#_ENREF_5))  *“International harmonisation of regulations would simplify international trade.”* ([Gupta et al. 2013](#_ENREF_29))  **Box 4c:**  *“Yet as long as regulatory agencies lack the immediate funds to research the implications of nanotechnology extensively on their own, they will need to pass the burden on to industry to build a coherent body of knowledge about these implications. But such requirements could easily exceed the amount that industry is generally willing to contribute. Such disagreement will undoubtedly be played out in the form of a power struggle between agencies and industry.”* ([Becker 2013](#_ENREF_3))  *“The main reason given by supporters of labeling was that the consumer has a right to know, with one subject declaring, ‘If it’s a nano-scale material, people should know, hands down.’’’* ([Becker 2013](#_ENREF_3))  *“Labeling is an unusually contentious issue for the domain of nanotechnology, with much disagreement about whether or not products containing nanomaterials should be labeled as such, and what information, if any, should be included on a label. The European Union has already enacted labeling requirements for nanotechnology ingredients in cosmetics. But in the United States, it is still undecided how much ought to be known before accurate labels can be produced. But what is perhaps most contentious is if the need for highly accurate labeling trumps the consumer’s ‘‘right to know’’, given that consumers are increasingly coming into contact with nano-enabled products. Still, the question may be posed, if only a vague label is given, what information do consumers really have?”* ([Becker 2013](#_ENREF_3))  *“In the present study, we tested consumers’ acceptance of hypothetical food concepts. The formulation of the scenario was not constrained by current regulations. Regulations are constantly changing. For middle or long term planning, industry and NGO’s should know under which conditions the public accepts nanotechnology in food products. Currently, the use of nanotechnology encapsulation methods does not have to be labeled in the USA or the EU. The case of GM food demonstrates, however, that pressure from interest groups may result in new regulations. GM food must be labeled in the EU and in Switzerland, for example. Labeling of nanotechnology food products is discussed in various countries (Burri and Bellucci, 2008). It is important for the food industry, therefore, to have some knowledge of the conditions under which nanotechnology is accepted by consumers. Otherwise, the food industry will not be well prepared for possible future regulations related to nanotechnology.”* ([Siegrist et al. 2009](#_ENREF_69))  **Box 4d:**  *“Respondents with high levels of trust perceived more beneﬁts associated with the nanotechnology applications compared with respondents with low levels of trust.”* ([Siegrist et al. 2008](#_ENREF_70))  *“Social trust (trust in sciences/consumer protection agencies) had a signiﬁcant effect on the perceived risks of nano-outside applications but had no effect on the perceived risk of nano-inside applications.”* ([Siegrist et al. 2008](#_ENREF_70))  **Box 4e:**  *“Familiarity with nanotechnology is found to play a role in accepting nanotechnology.”* ([Bieberstein et al. 2013](#_ENREF_6))  *“Consumer choice and the right to be informed were reasons for desiring the label and were typically invoked in these exchanges. The label therefore acted as an enabler of consumer choice from their perspective.”* ([Brown and Kuzma 2013](#_ENREF_11))  **Box 4f:**  *“… commercializers interviewed here focused on carrying out subjective risk/beneﬁt analyses by performing in-house testing and utilizing common sense to come to an understanding of the risks.”* ([Becker 2013](#_ENREF_3))  **Box 4g:**  *“However, even though they were not familiar with the technology behind the products, they were not scared. In contrast, grasping their own boundaries can foster interest in and fascination with nanotechnology.”* ([Simons et al. 2009](#_ENREF_71))  *“The more that negative affect and the less that control was associated with a nanotechnology food application or nanotechnology food packaging, the higher the perceived risk….The more that negative affect and the less that control was associated with a nanotechnology food application, the lower the perceived beneﬁt.”* ([Siegrist et al. 2008](#_ENREF_70))  *“Skepticism about their ineffectual nature stemmed from concerns about correctly interpreting a label or that labels simply do not motivate behavioral change…” (Brown et al. 2015)* |
| --- |

**Supplementary** **Data 8: Box 5 Quotations to illustrate the ‘Characteristics of food nanotechnology’ theme**

| **Box 5a:**  *“In sum, people who preferred natural and healthy food associated more risks and fewer benefits with nanotechnology food products compared to people who did not put emphasis on those food qualities.”* ([Stampfli et al. 2010](#_ENREF_72))  *“…consumers are more sensitive to technologies directly modifying the product.”* ([Marette et al. 2009](#_ENREF_40))  **Box 5b:**  *“Experts were of the opinion that people will distinguish between applications on the basis of the personal advantages that would accrue to an individual, and how real or close to reality these applications will appear to the public.”* ([Gupta et al. 2012](#_ENREF_30))  *“For example, nanotechnology is promoted widely as a technological solution to enhance food security, which is a more pressing problem in the developing world…”* ([Gupta et al. 2013](#_ENREF_29))  *“More speciﬁcally, participants were most willing to use nanotechnology food packaging for the beneﬁcial functions of enhancing nutrition…, reducing spoilage …, and leading to cheaper production...”* ([Brown and Kuzma 2013](#_ENREF_11)) |
| --- |

**Supplementary** **Data 9: Box 6 Quotations to illustrate the ‘Link to historical agri-food technology concerns’ theme**

| **Box 6:**  *“We can show that a high- risk perception of GM food correlates with lower WTP* [willingness to pay] *of nano-food and nano-packaging, both in France and in Germany.”* ([Bieberstein et al. 2013](#_ENREF_6))  *“It was assumed that a new, still unknown technology with high levels of uncertainty, as is the case for nanotechnology food applications, may make consumers rely on previous evaluations of other already known food technologies, such as genetic modiﬁcation in food. In both countries and for both products, higher risk judgements of GM food are linked to a signiﬁcantly lower WTP for the nano-food and nano-packaging.”* ([Bieberstein et al. 2013](#_ENREF_6))  *“In the interview, “negative public perceptions” were a particular concern due to misinformation and “bad press” from comparisons to GM foods. There were fears that misinformation could result in mistrust by the consumers, which in turn could have serious im- plications for the agri-food industry, like in the recent example of the horsemeat scare. This was replicated in the survey, with the main challenges regarding the use of nanotechnology in agri-food being “information and knowledge deﬁcits”, “public acceptance”, and “long term health implications” (Handford et al. 2015)* |
| --- |

**Supplementary** **Data 10: Box 7 Quotations to illustrate the ‘Marketing and commercialisation’ theme**

| **Box 7a:**  *“In the area of promotion, consumers must be informed of the risks and beneﬁts associated with nanotechnology, as the public appreciates receiving information that can facilitate the decision to buy traditionally produced foods or foods produced with new technologies...”* ([Schnettler et al. 2013b](#_ENREF_62))  *“This indicates that the brand helps reduce uncertainty and the perception of risk when purchasing foods produced with new technologies such as GM and nanotechnology.”* ([Schnettler et al. 2013a](#_ENREF_60))  **Box 7b:**  *“Increase number of consumers that are purchasing or consuming agrifood nanotechnology products will ultimately lead to increase R&D* [research and development] *investment rate, more products in R&D, increase in the rate of commercialization, and more agrifood products on the market.”* ([Yawson and Kuzma 2010](#_ENREF_82))  *“…that people would expect water ﬁltration and food packaging to be commercialised sooner than most other applications.”* ([Gupta et al. 2013](#_ENREF_29))  *“Assuming that experts shape the process of innovation, one might anticipate that the ﬁrst products introduced into the (European) market will be those which experts perceive will be viewed as most beneﬁcial and least related to societally less acceptable application in, for example, the agrifood sector.”* ([Gupta et al. 2012](#_ENREF_30)) |
| --- |

**Supplementary Data 11: Box 8 Quotations to illustrate the ‘Future applications of agri-food nanotechnology’ theme**

| **Box 8a:**  *“Future studies may wish to examine how consumers react to different descriptions of nanotechnology. Moreover, further research should identify factors that augment or hinder the acceptance of nanotechnology foods and should also examine possible cultural differences.”* ([Siegrist et al. 2009](#_ENREF_69))  *“Comparison between expert and public opinion is therefore needed in order to determine whether what is technically possible from implementation enabling technologies such as nanotechnology aligns with societal preferences.”* ([Gupta et al. 2013](#_ENREF_29))  **Box 8b:**  *“Future studies may wish to examine how consumers react to realistic nanotechnology foods.”* ([Siegrist et al. 2007](#_ENREF_67))  *“However, these results suggest that when investigating the acceptance of nanotechnology applications, a large number of consumer-related variables should be considered, such as their psychographic and psychological characteristics, and should not be conﬁned solely to their demographic characteristics.”* ([Schnettler et al. 2014](#_ENREF_61))  **Box 8c:**  *“This suggests that experts speculate that social negativity will arise as nanotechnology is commercialised, in particular within the agrifood sector, and that at this stage in implementation understanding why this occurred with genetic modiﬁcation may be useful when determining how nanotechnology might be commercialised.”* ([Gupta et al. 2012](#_ENREF_30))  *“Future research could adopt a more nuanced focus both on application domain and the social contexts in which they will be encountered and understood by social groups and persons in different social locations.”* ([Conti et al. 2011](#_ENREF_18))  *“In step with most of the past research, we investigated willingness to buy new food products and not the actual behavior. Respondents did not taste the food. Results of past studies suggest that taste is an important factor inﬂuencing consumers’ willingness to use functional foods (Verbeke, 2006). Future studies may wish to examine how consumers react to different descriptions of nanotechnology.”* ([Siegrist et al. 2009](#_ENREF_69))  **Box 8d:**  *“Handling public education of different stakeholder groups, public engagement in the governance and regulatory process, and involvement of consumers in proactive debate on risks and benefits of agrifood nanotechnology.”* ([Yawson and Kuzma 2010](#_ENREF_82))  *“Public engagement has a dual role in consumer acceptance of agrifood nanotechnology and public engagement will lead to increased consumer awareness which will enable consumer acceptance or rejection of agrifood nanotechnology to be based more on facts than on suspicions or speculative claims and engaging the public will enhance the depth of interaction and confidence and trust among those involved in the research, development, governance, and regulation of agrifood nanotechnology, the public, and NGOs (Mantovani et al. 2009). This is crucial if satisfactory trade-offs of risks and benefits of agrifood nanotechnology are to be defined appropriately.”* ([Yawson and Kuzma 2010](#_ENREF_82))  *“So it is crucial to involve trusted agencies and even speciﬁed NGOs in risk communication process. Sooner or later bad news on nanotechnology will become available for the uninformed general public, so it is wisdom to take risk communication actions as soon as possible.”* ([Farshchi et al. 2011](#_ENREF_22))  *“Special emphasis ought to be given to transparency and accountability in communication.”* ([Köhler and Som 2008](#_ENREF_35)) |
| --- |
